# Tirzepatide in recurrent hypertriglyceridemia-mediated pancreatitis and type 2 diabetes: a case report

**DOI:** 10.1186/s40842-026-00318-z

**Published:** 2026-08-01

**Authors:** Benjamin Lechner, Katharina Lechner, Ines Freibothe, Anja Vogt

**Affiliations:** 1 Department of Medicine IV, LMU University Hospital, LMU Munich, Munich, Germany; 2 DZHK (German Centre for Cardiovascular Research), Partner Site Munich Heart Alliance, Munich, Germany; 3 TUM School of Medicine and Health, Technical University of Munich, Munich, Germany; 4 Helmholtz Munich, Institute of Epidemiology, Munich, Germany

**Keywords:** Hypertriglyceridemia, Acute pancreatitis, Tirzepatide, Type 2 diabetes, Metabolic dysfunction, MASLD

## Abstract

**Background:**

Hypertriglyceridemia‑mediated acute pancreatitis (HTG‑AP) is a clinically relevant complication of severe metabolic dysfunction, yet incretin‑based therapy is often withheld because current recommendations discourage its use in patients with a history of pancreatitis.

**Case presentation:**

We report on a woman born in 1993 with severe hypertriglyceridemia known since 2018, type 2 diabetes diagnosed in 2022, recurrent HTG-AP from 2023 onward, chronic fibrosing pancreatitis, and severe metabolic dysfunction-associated steatotic liver disease (MASLD). Despite lifestyle measures, fenofibrate, high-dose omega-3 fatty acids, and intensified insulin therapy, she presented again in March 2025 with acute pancreatitis and triglycerides of 4.400 mg/dL. After stabilization of acute pancreatitis and marked triglyceride reduction due to prolonged fasting and intensified insulin therapy, tirzepatide was started on March 27, 2025. At initiation, triglycerides were 335 mg/dL and HbA1c was 7.0%. During follow-up, insulin was fully discontinued, triglycerides normalized to 137 mg/dL at 3 months and 85 mg/dL at 12 months, HbA1c improved to 5.5%, inflammatory markers normalized, and no further pancreatitis episodes occurred.

**Conclusion:**

This single-patient case observation illustrates that tirzepatide may be considered on an individualized basis in selected cases of metabolically driven pancreatitis, particularly when metabolic dysfunction is presumed to be the dominant driver. However, the favorable clinical course observed in this case represents an association only and does not establish causality.

## Background

Hypertriglyceridemia-mediated acute pancreatitis (HTG-AP) is an important cause of pancreatitis, accounting for approximately 1 to 10% of cases and often occurring in the setting of diabetes and metabolic dysfunction [1]. Concerns about incretin-based therapies and pancreatitis have led to cautious use in patients with a history of pancreatitis, although larger contemporary analyses have not shown a consistent increase in pancreatitis risk with GLP-1 receptor agonists or tirzepatide compared with other glucose-lowering agents [2–4]. We report on a young woman with severe recurrent HTG-AP, type 2 diabetes, chronic pancreatic injury, and severe metabolic dysfunction in whom tirzepatide initiation was associated with sustained metabolic improvement and 12 months without further pancreatitis recurrence.

## Case presentation

A woman born in 1993 was first found to have marked hypertriglyceridemia in 2018 as an incidental laboratory finding, with triglycerides of 1,300 mg/dL. At that time, her body mass index (BMI) was 22 kg/m² and her only regular medication was an inhaled formoterol and budesonide combination for asthma. Concomitant testing showed a HbA1c of 5.8%, a HOMA-IR of 2.6, and a Matsuda index of 3.7, indicating liver and whole-body insulin resistance without overt diabetes.

Lifestyle modification and fenofibrate therapy were initiated, but triglyceride levels remained highly variable, ranging from 200 to 2,000 mg/dL during follow-up. There was no relevant alcohol use, estrogen therapy, or other obvious secondary cause of severe hypertriglyceridemia. In 2022, diabetes mellitus was diagnosed with a HbA1c of 6.8%. Diabetes-associated autoantibodies were negative and C-peptide levels were elevated, supporting type 2 diabetes rather than autoimmune diabetes. Metformin was started but later discontinued because of intolerance.

From 2023 onward, the patient experienced recurrent events of acute pancreatitis associated with very high triglyceride levels and concomitant hospitalization, with one event in 2023 and two further events in 2024. Because of metformin intolerance and the need for reliable glycemic control, an intensified conventional insulin regimen with basal insulin glargine U300 and prandial insulin lispro (0.3–0.4 IU/kg/day) was started. Despite continued treatment with fenofibrate 250 mg daily and high-dose omega-3 fatty acids at 4 g/day, triglyceride levels remained poorly controlled.

In early 2025, the patient presented to our hospital with upper abdominal pain suggesting another pancreatitis. On admission (March 3, 2025), triglycerides peaked at 4,400 mg/dL and C-reactive protein reached 28.8 mg/dL. Acute pancreatitis was confirmed, representing the fourth episode of symptomatic HTG-AP. At that time, the patient’s BMI was 24 kg/m² and the HbA1c 6,9%. Further imaging and clinical assessments supported the diagnoses of chronic fibrosing pancreatitis and severe metabolic dysfunction-associated steatotic liver disease (MASLD). During hospitalization, triglycerides decreased rapidly from 4,400 mg/dL to 335 mg/dL under prolonged fasting and intensified intravenous and subcutaneous insulin therapy, while fenofibrate and high‑dose omega‑3 fatty acids were continued. This acute improvement occurred before initiation of tirzepatide and reflects the expected effect of strict caloric restriction and intensive insulin treatment in severe hypertriglyceridemia.

Comprehensive genetic testing was performed to evaluate two separate diagnostic questions. First, hereditary pancreatitis was excluded by targeted Sanger sequencing after specific PCR amplification of PRSS1, SPINK1, CTRC, and the CEL-HYB allele, revealing no pathogenic variants. Second, a monogenic cause of severe hypertriglyceridemia was evaluated using the Synlab combined hyperlipoproteinemia next-generation sequencing (NGS) panel; no pathogenic or likely pathogenic variants were identified. Copy number variations (CNVs) were also assessed and showed no evidence of exon-spanning deletions or duplications in the analyzed genes. Notably, the Synlab panel identified a homozygous APOA5 *3 haplotype (p.Ser19Trp), classified as a risk factor rather than a pathogenic variant, with a minor allele frequency of 0.1–15% depending on ethnicity. This variant has been associated with susceptibility to hypertriglyceridemia and late-onset hyperchylomicronemia and may have contributed to the patient’s severe triglyceride phenotype in the context of additional metabolic risk factors. Physical examination showed preserved subcutaneous adipose tissue without regional lipoatrophy. The patient’s overall phenotype was marked by relative sarcopenia with predominant abdominal adiposity, rather than a classical lipodystrophic pattern. Given the recurrent HTG-AP, chronic pancreatic damage without impaired insulin secretion (i.e. normal C-Peptide levels), persistent metabolic dysfunction, and the burden of intensified insulin therapy tirzepatide was started on March 27, 2025 and titrated to 5 mg once weekly. Laboratory values at tirzepatide initiation showed triglycerides of 335 mg/dL, HbA1c 7.0%, C-reactive protein 0.9 mg/dL, alanine aminotransferase (ALT) 32 U/L, and gamma-glutamyl transferase (GGT) 55 U/L. Over the subsequent three months, prandial insulin lispro and basal insulin glargine U300 were gradually tapered and fully discontinued by June 18, 2025 without deterioration in glycemic control.

By June 18, 2025, triglycerides had fallen to 137 mg/dl, HbA1c to 6.3%, and C-reactive protein to 0.7 mg/dL, allowing discontinuation of fenofibrate and omega-3 fatty acids on the same date. After six months of tirzepatide therapy (September 2025), the patient had lost approximately 10 kg (BMI 20 kg/m²), triglycerides had fallen to 177 mg/dL, HbA1c 5.8%, C-reactive protein 0.6 mg/dL, ALT 39 U/L, and GGT 42 U/L. After one year (February 2026), the patient had lost no further weight, stabilizing at a BMI of 20 kg/m², fasting triglycerides 85 mg/dL, and HbA1c 5.5%. C-reactive protein normalized to 0.4 mg/dL. Liver enzymes rose mildly and transiently at three months (ALT 85 U/L, GGT 79 U/L) before normalizing by 12 months (ALT 26 U/L, GGT 31 U/L). Rosuvastatin 5 mg daily was started before the last assessment because carotid atherosclerotic plaques were detected. No further episodes of pancreatitis occurred, and the patient reported marked improvement in quality of life.

## Discussion

This case is notable for recurrent HTG-AP despite lifestyle measures, fenofibrate, high-dose omega-3 fatty acids, and intensified insulin therapy, together with a negative work-up for hereditary pancreatitis, monogenic hypertriglyceridemia, or lipodystrophy. The phenotype also stands out because severe metabolic dysfunction occurred despite a normal BMI of only 22 to 24 kg/m², underscoring once more that clinically relevant metabolic pancreatitis or more broadly, metabolic dysfunction, can occur within normal BMI ranges. However, the homozygous *APOA5* *3 haplotype identified in our patient represents a well-established polygenic risk factor for hypertriglyceridemia and hyperchylomicronemia, which in combination with type 2 diabetes, insulin resistance, and metabolic dysfunction likely contributed to the severe triglyceride phenotype. Although no regional lipoatrophy was observed and clinical examination showed preserved subcutaneous adipose tissue with relative sarcopenia and predominant abdominal adiposity, a partial lipodystrophy syndrome cannot be definitively excluded. Formal body-composition assessments, including DEXA, skinfold measurements, and serum leptin levels, were not performed.

HTG-AP is well recognized when triglyceride levels are markedly elevated and type 2 diabetes itself is associated with a higher risk of acute pancreatitis independent of classical risk factors [1, 5–8]. In such patients, pancreatic injury likely reflects a broader metabolic milieu that includes insulin resistance, severe triglyceride excursions, ectopic fat, and systemic inflammation rather than a single isolated trigger [9–11].

**Fig. 1:**
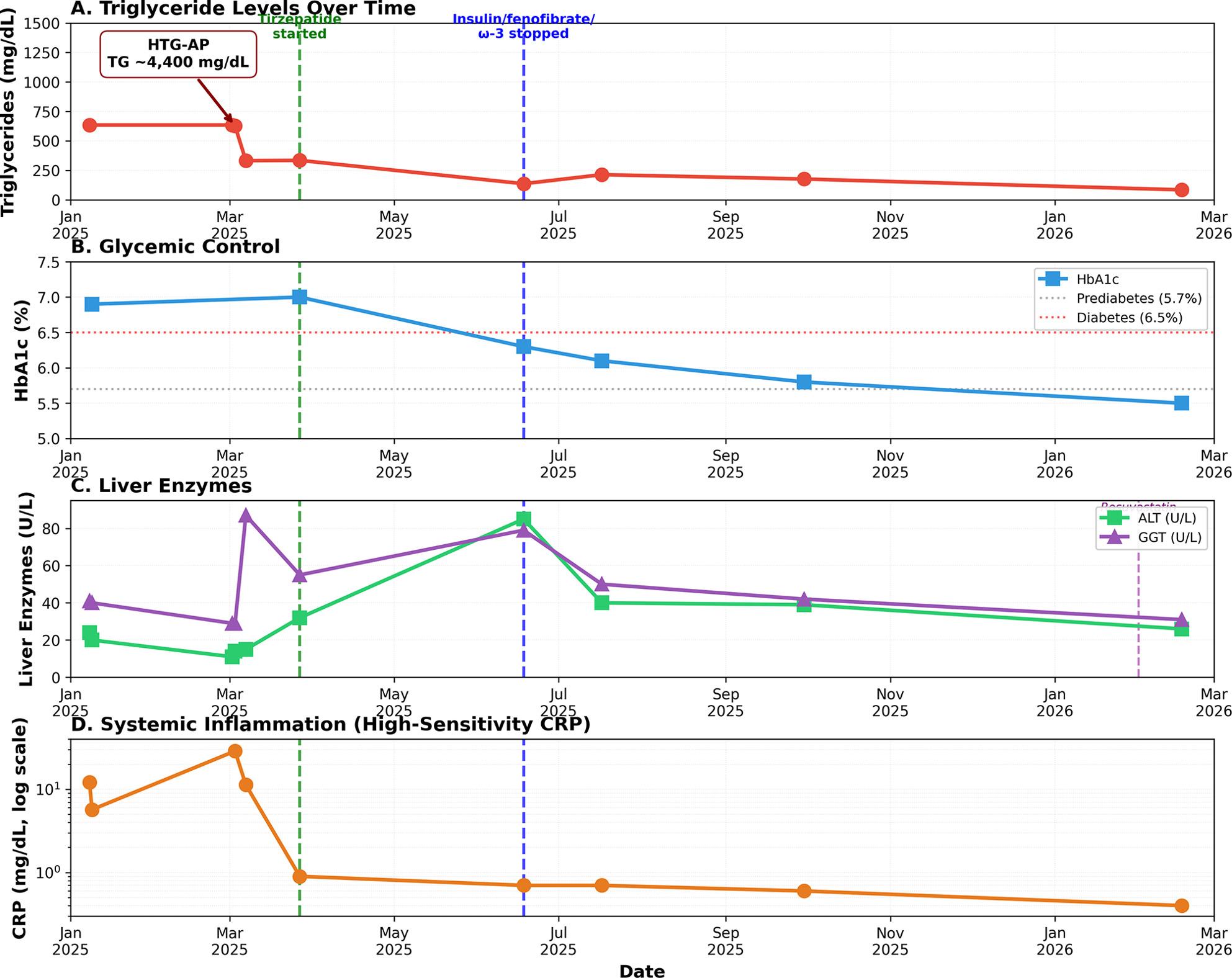
Metabolic response after tirzepatide initiation in recurrent hypertriglyceridemia-mediated pancreatitis. (**A**) Triglyceride levels over time with key therapy changes. Peak triglycerides reached 4.400 mg/dL during the index episode of acute pancreatitis in early March 2025. Tirzepatide initiation on March 27, 2025 was followed by further triglyceride reduction from 335 to 137 mg/dL within three months and to 85 mg/dL at 12 months. (**B**) HbA1c trajectory showing improvement from 7.0% at tirzepatide initiation to 5.5% at 12 months. (**C**) Liver enzymes showing a transient increase at three months followed by normalization by 12 months. (**D**) C-reactive protein showing marked inflammation during acute pancreatitis and normalization during follow-up

The chronological sequence in this case supports a beneficial association between tirzepatide and subsequent metabolic stabilization in the absence of obesity. The marked decrease of triglycerides from 4.400 mg/dL during the index pancreatitis to 335 mg/dL occurred during acute management and recovery before tirzepatide initiation. However, after tirzepatide was started, triglycerides normalized to 137 mg/dL at three months and 85 mg/dL at 12 months, glycemic control improved substantially, insulin was discontinued without diabetes worsening, and no further pancreatitis episodes occurred during 12 months of follow-up (see Table 1; Fig. 1). These changes persisted despite later withdrawal of fenofibrate and omega-3 fatty acids. Following initiation of tirzepatide 5 mg once weekly, BMI decreased from 24 kg/m² at admission to approximately 20 kg/m² within 6 months, after which body weight remained stable without further decline during follow-up. Given the weight stabilization and the favorable metabolic response, we considered the continued use of tirzepatide at this dose to be justified under regular clinical weight monitoring. In case of further weight loss, we plan to consider cautious dose reduction (e.g. to 2.5 mg) or a supervised discontinuation regime in the future, combined with close monitoring of triglycerides and glycemic control. To optimize body composition during weight loss regular dietary counseling to ensure adequate protein and micronutrient intake was performed and resistance exercise was recommended.

**Table 1:** Key laboratory values over time

Time Point	Triglycerides (mg/dL)	HbA1c (%)	CRP (mg/dL)	ALT (U/L)	GGT (U/L)
Baseline (2018)	1,300	5.8	—	—	—
Pre-tirzepatide (Jan 2025)	635	6.9	12.1	24	41
Tirzepatide start (Mar 27, 2025)	335	7.0	0.9	32	55
Insulin, fibrate, and omega-3 stop (Jun 18, 2025)	137	6.3	0.7	85	79
6 months (Sep 2025)	177	5.8	0.6	39	42
1 year (Feb 2026)	85	5.5	0.4	26	31

Peak triglycerides of 4.400 mg/dL occurred during the index pancreatitis episode on March 3, 2025 and are described in the text and figure legend rather than as a serial outpatient time point

Importantly, these observations must be interpreted with caution because a single case cannot establish whether tirzepatide prevented recurrence of hypertriglyceridemia-mediated pancreatitis. Nevertheless randomized trials and meta-analyses have not shown a consistent pancreatitis signal for GLP-1 receptor agonists or tirzepatide [2–4, 12, 13], and tirzepatide improves glycemia, body weight, and triglycerides in patients with type 2 diabetes independent of body weight [14–16]. In patients whose pancreatic injuries appear to be driven predominantly by metabolic dysfunction, concerns partly driven by confounding by indication should not automatically preclude incretin-based therapy. In support of our observation, more recent data suggests a decreased risk of acute pancreatitis in people with type 2 diabetes and tirzepatide or semaglutide treatment and a history of acute pancreatitis [13].

## Conclusion

In this patient with type 2 diabetes and recurrent HTG-AP, tirzepatide initiation was associated with sustained improvement in triglycerides, glycemia, inflammatory markers, and 12-month recurrence-free follow-up. This case supports, in line with emerging literature, careful, individualized consideration of incretin-based therapy in metabolically driven pancreatitis rather than automatic exclusion based solely on prior pancreatitis history. Metabolic pancreatitis related to diabetes and obesity may be underrecognized and concerns partly driven by confounding by indication should not automatically preclude carefully monitored incretin-based therapy when metabolic dysfunction appears to be the dominant contributor to pancreatic injury.

This report has several limitations. First, as a single-case observation, it cannot establish whether tirzepatide therapy contributed causally to the prevention of recurrent pancreatitis. Second, formal body-composition assessments, including DEXA, skinfold-thickness measurements, and serum leptin levels, were not performed; therefore, partial lipodystrophy cannot be definitively excluded despite the absence of overt regional lipoatrophy on clinical examination and negative genetic testing.

## Data Availability

No datasets were generated or analysed during the current study.

## Abbreviations

ALT: alanine aminotransferase
APOA5: apolipoprotein A5
BMI: body mass index
CEL-HYB: carboxyl-ester-lipase-hybride allele
CNV: copy number variation
CTRC: Chymotrypsin C
GLP-1: glucagon-like peptide-1
GGT: gamma-glutamyl transferase
HOMA-IR: homeostatic model assessment of insulin resistance
HTG-AP: Hypertriglyceridemia-mediated acute pancreatitis
MASLD: metabolic dysfunction-associated steatotic liver disease
NGS: next-generation sequencing
PCR: polymerase chain reaction
PRSS1: protease serin 1
SPINK1: serine peptidase inhibitor kazal-type 1
